# Diagnostic accuracy of nucleic acid amplification tests (NAATs) in urine for genitourinary tuberculosis: a systematic review and meta-analysis

**DOI:** 10.1186/s12879-017-2476-8

**Published:** 2017-06-05

**Authors:** Carlos Altez-Fernandez, Victor Ortiz, Majid Mirzazadeh, Luis Zegarra, Carlos Seas, Cesar Ugarte-Gil

**Affiliations:** 1 Facultad de Medicina Alberto Hurtado, Universidad Peruana Cayetano Heredia, Av. Honorio Delgado 430, Urb. Ingeniería, S.M, P Lima, Perú; 2 Department of Urology, Wake Forest University, Winston Salem, NC USA; 3 Servicio de Urología, Hospital Nacional Cayetano Heredia, Lima, Perú; 4 Instituto de Medicina Tropical Alexander von Humboldt, Universidad Peruana Cayetano Heredia, Lima, Peru; 5 Departamento de Enfermedades Infecciosas, Tropicales y Dermatológicas, Hospital Nacional Cayetano Heredia, Lima, Peru; 6 Department of Clinical Research, London School of Hygiene and Tropical Medicine, London, UK; 7 Department of International Health, Johns Hopkins Bloomberg School of Public Health, Baltimore, USA

**Keywords:** Genitourinary tuberculosis, Nucleic acid amplification test, Systematic review

## Abstract

**Background:**

Genitourinary tuberculosis is the third most common form of extrapulmonary tuberculosis. Diagnosis is difficult because of unspecific clinical manifestations and low accuracy of conventional tests. Unfortunately, the delayed diagnosis impacts the urinary tract severely. Nucleic acid amplification tests yield fast results, and among these, new technologies can also detect drug resistance. There is lack of consensus regarding the use of these tests in genitourinary tuberculosis; we therefore aimed to assess the accuracy of nucleic acid amplification tests in the diagnosis of genitourinary tuberculosis and to evaluate the heterogeneity between studies.

**Methods:**

We did a systematic review and meta-analysis of research articles comparing the accuracy of a reference standard and a nucleic acid amplification test for diagnosis of urinary tract tuberculosis. We searched Medline, EMBASE, Web of Science, LILACS, Cochrane Library, and Scopus for articles published between Jan 1, 1990, and Apr 14, 2016. Two investigators identified eligible articles and extracted data for individual study sites. We analyzed data in groups with the same index test. Then, we generated pooled summary estimates (95% CIs) for sensitivity and specificity by use of random-effects meta-analysis when studies were not heterogeneous.

**Results:**

We identified eleven relevant studies from ten articles, giving information on PCR, LCR and Xpert MTB/RIF tests. All PCR studies were “in-house” tests, with different gene targets and had several quality concerns therefore we did not proceed with a pooled analysis. Only one study used LCR. Xpert studies were of good quality and not heterogeneous, pooled sensitivity was 0·87 (0·66–0·96) and specificity was 0·91 (0·84–0·95).

**Conclusion:**

PCR studies were highly heterogeneous. Among Xpert MTB/RIF studies, specificity was favorable with an acceptable confidence interval, however new studies can update meta-analysis and get more precise estimates. Further high-quality studies are urgently needed to improve diagnosis of genitourinary tuberculosis.

**Protocol registration:**

PROSPERO CRD42016039020.

**Electronic supplementary material:**

The online version of this article (doi:10.1186/s12879-017-2476-8) contains supplementary material, which is available to authorized users.

## Background

Tuberculosis (TB) is still one of the world’s biggest threats to public health. In 2014, TB killed 1.5 million people and was the single infectious disease leading cause of death worldwide [1]. The World Health Organization (WHO) has estimated that only in 2014 there were 9·6 million new TB cases, 12% of which were HIV-positive [1].

Extrapulmonary TB (EPTB) accounts for approximately 10 to 20% of all the TB affected population. Genitourinary TB (GUTB) is the third most common location of EPTB, after pleural and lymph node involvement [2]. In addition, concomitant GUTB and pulmonary TB is found in 2–10% and in 15–20% of patients in developed and developing countries, respectively [2].

GUTB is defined as the infection by *Mycobacterium tuberculosis* of the urinary tract, the male genitalia or the female genitalia, however, most authors refer to GUTB for reporting only the first [3–5]. The infection occurs in most cases after long periods of latent infection followed by hematogenous spread to the kidneys, epididymis or fallopian tubes; prostate seeding has also been reported but is extremely rare. Other genitourinary organs are affected by local spread [5–7].

Clinical manifestations of GUTB are non-specific, depending on the organs affected and the severity of their involvement, and can mimic several urologic and gynecologic diseases [8]. Diagnosing GUTB is difficult and often overlooked. Conventional diagnostic tests include urine microscopy and culture. Since GUTB is usually paucibacillary [9], most samples evaluated by microscopy are negative. Although culture is the gold standard for diagnosing GUTB, currently available culture methods, including both solid and liquid platforms are insensitive for diagnosing GUTB, and show a number of disadvantages including long-turn around times, high contamination rates and cost [10].

Furthermore, delayed diagnosis may result in worse outcomes, such as infertility, non-functioning unilateral kidney, contracted bladder and even renal failure [8]. Case series reported that 26·9% of patients on average had a non-functioning unilateral kidney, 7.4%, renal failure and 10% had contracted bladder at the time of diagnosis of genitourinary tuberculosis [2, 4].

Nucleic acid amplification tests (NAATs), have been developed to overcome the limitations of conventional tests in TB [11–15]. These can yield results in between 2 to 48 h [16] NAATs can be classified as commercial or in-house (“home-brew”) tests [16] They can be classified, as well, by their mechanism: polymerase chain reaction (PCR) tests, ligase chain reaction(LCR) tests and more recent technologies (variants of PCR) such as Xpert MTB/RIF and Genotype® MTBDRplus [17, 18].

While the use of NAAT has been well studied in pulmonary TB, their use in GUTB lacks enough high-quality evidence [11, 19, 20]. Therefore, we conducted this systematic review to determine the accuracy of NAAT performed in urine for diagnosing GUTB and to identify the factors related to heterogeneity between studies of the result.

## Methods

We developed our study in accordance with the Cochrane’s group guidelines and methods for systematic reviews and meta-analyses of diagnostic tests [21]. The protocol was registered at PROSPERO database with the number CRD42016039020.

### Search strategy and selection criteria

We searched Medline, EMBASE, Scopus, Lilacs, Cochrane Library and Web of Science for studies in all languages published from January 1, 1990, to April 14, 2016 (see Additional file 1: Appendix S1 for search terms).

We included all the studies that compared a NAAT in urine for diagnosis of GUTB with the gold standard. These studies should have presented data in the form of true positives (TP), true negatives (TN), false positives (FP) and false negatives (FN) In case insufficient data were reported, authors were contacted via email to provide additional information regarding their published reports.

We included studies that used a microbiological or a broad standard reference as gold standard. We define the microbiological standard reference as a positive mycobacterial culture on solid or liquid media for one or more specimens from each patient. We define a broad standard reference as either a microbiological standard reference or clinical manifestations of the disease with clinical response after appropriate treatment. We excluded 3 studies that showed results fewer than ten patients, as such results are at high risk of bias. A total of 13 patients were excluded using this criterion. We also excluded studies that had a case-control design, as these may lead to overestimation of accuracy in diagnostic studies.

**Fig. 1:**
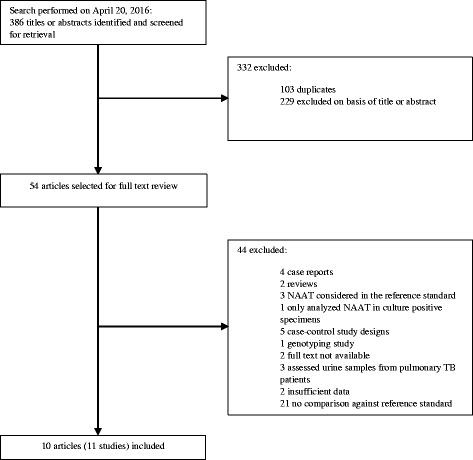
Selection of studies reporting on the use of a NAAT for GUTB diagnosis in urine

Two reviewers (CA and VO) independently screened the collected citations for relevance and revised full-text articles with pre-specified eligibility criteria; disagreements about study selection were resolved by consultation with a third reviewer (CU-G). The results of the literature search are presented in a flowchart following the PRISMA guidelines (Fig. 1) [22].

### Data extraction and quality appraisal

CA and VO extracted data and resolved disagreements by consensus (Additional file 1). We assessed study quality with the Quality Assessment Tool for Diagnostic Accuracy Studies (QUADAS-2) [23]. QUADAS-2 consists of four domains: patient selection, index test, reference standard, and flow and timing. We assessed all domains for the potential for risk of bias and the first three domains for concerns regarding applicability. We used questions, called signaling questions, for each domain to form judgments about the risk of bias. As recommended, we first developed guidance on how to evaluate each signaling question and interpret this information tailored to this review. Then, one review author (CA) piloted the tool with four of the included studies. Based on experience gained from the pilot study, we finalized the tool (see Additional file 1: Appendix S2 for quality appraisal). Two authors (CA and VO) independently assessed the methodological quality of the included studies with the finalized tool; disagreements about methodological quality were resolved by consultation with a third reviewer (CU-G).

### Statistical analysis

We used standard methods recommended for meta-analyses of diagnostic studies [21] Analyses were done using the R statistical programming language version 3 [24, 25].

To minimize projected heterogeneity, we decided a priori to analyze data in certain subgroups: studies using PCR, studies using LCR and studies using Xpert MTB/RIF. For each subgroup, we assessed heterogeneity visually with forest plots and summary receiver-operating characteristic (SROC) curves with 95% prediction regions, and statistically with χ^2^ statistics. Regarding the forest plots, we used a statistic continuity correction of 5% applied to the reported sensitivity and specificity. This correction affected studies with large confidence intervals and extreme values, we then used these results for further analysis as recommended for meta-analyses of diagnostic studies [21]. We generated pooled summary estimates and differences for sensitivity and specificity with 95% confidence intervals by using a bivariate summary ROC curve [26]. It is assumed that sensitivity and specificity vary across studies because of differences in study populations, sampling errors, and differences in implicit thresholds applied to the data to separate patients. Thus, a random-effect model was applied to account for between-study heterogeneity.

## Results

We identified 386 citations, of which 54 were potentially relevant based on the title and abstract (Fig. 1). After full-text review, ten articles were included in our analysis, providing data of eleven studies addressing different NAATs.

### Study characteristics

Of the eleven studies selected, eight studies used PCR, one study used LCR and, two studies used Xpert MTB/RIF. All of the PCR studies were “in-house” tests. The average size of each study was 248 (specimens or subjects), with a range of 20 to 1000. Table 1 outlines the main characteristics of these studies.

**Table 1:** Description of studies in the systematic review and reported measures of test accuracy

Study	Year	Country	Prospective data collection	Double or single blinding	Index test used	Specific details of index test used	Number of patients	Specimens per patient	Reference standard	Reported sensitivity	Reported specificity
Khan et al. [41]	2013	Pakistan	Yes	No	In-house	Real Time PCR IS6110, MPB-64, 16rRNA	50	3 CMUS	A	88·6	96·5
Garcia-Elorriaga et al. [42]	2009	Mexico	No	No	In-house	Nested PCR 32-kDa, MTP40 and IS6110	20	1	a	100	82
Khosravi et al. [43]	2010	Iran	Yes	No	In-house	Nested PCR IS6110	200	1	A	100	100
Raghavendran et al. [44]	2016	India	Yes	No	In-house	PCR (gene target nor reported)	48	1	A	89·5	89·6
Hemal et al. [45]	2000	India	Yes	No	In-house	PCR MPB-64	42	Unknown	b	94·3	85·7
van Vollenhoven et al. [46]	1996	South Africa	Yes	No	In-house	PCR M13 mp8	82	Unknown	A	100	100
Moussa et al. [47]	2000	Egypt	Yes	No	In-house	PCR 16S rRNA	1000	3 CMUS	A	87·05	98·9
Moussa et al. [47]	2000	Egypt	Yes	No	In-house	PCR IS6110	1000	3 CMUS	A	95·59	98·11
Gamboa et al. [48]	1998	Colombia	Yes	No	Commercial	LCx M. Tuberculosis Assay	69	Unknown	A	70	100
Hillerman et al. [49]	2011	Germany	Yes	Yes	Commercial	Xpert MTB/RIF	91	1	A	100	98.6
Tortoli et al. [50]	2012	Italy	Yes	Yes	Commercial	Xpert MTB/RIF	130	1	B	87·5	99·1

*CMUS* continuous day- morning urine sample, *A* microbiological reference standard (positive culture), *B* broad reference standard (either a positive culture or clinical manifestations with adequate treatment response after a minimum one-month follow-up); ^a^Final Physician Decision considering culture and treatment response; ^b^advanced and typical radiologic findings, positive urine smear or culture, and histologic examination of a biopsy or surgically resected specimen

Overall, nine studies were done in middle-income countries were TB burden is high. Only one study collected data retrospectively. Furthermore, a microbiological reference standard was used in eight studies, one used a broad reference standard and the other two used different combinations of clinical manifestations and culture.

Each of the studies that used PCR had a unique protocol for running the test. Six studies used a single PCR technique and two used a nested PCR technique; additionally, the gene target and primers were also different in each one.

### Quality appraisal

**Fig. 2:**
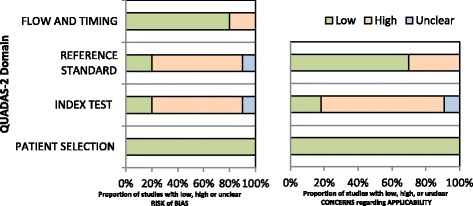
Summary of Quality Assessment tool for Diagnostic Accuracy tests (QUADAS-2). The point estimates of sensitivity and specificity from each study are shown as solid circles. Error bars are 95% confidence intervals. A statistical continuity correction of 5% has been applied

Figure 2 shows the results of the quality evaluation using the QUADAS-2 tool; 64% of the studies, all of which used PCR, were considered to be at high risk of bias in the index test and reference standard domains (see Additional file 1: Appendix S2 and Table S1 for detailed information). Within the index test and reference standard domain, studies were considered to be at high risk of bias because they did not blind operators to results of the reference standard or index test respectively, furthermore the protocols used in the “in-house” NAATs were not standardized and variations in technical aspects could have introduced bias.

For this latter reason, all the PCR studies were also considered as having a high concern of applicability in the index test domain. Additionally, 3 studies were considered as high concern of applicability in the reference standard domain because they used other than microbiological or broad reference standard.

### Summary measures

**Fig. 3:**
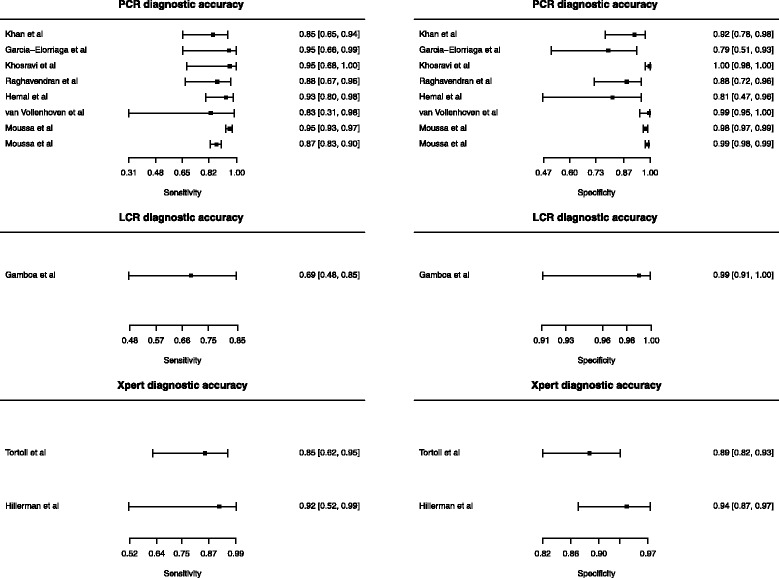
Forest plots of the diagnostic accuracy of PCR, LCR and Xpert MTB/RIF for the diagnosis of GUTB. Each triangle represents a study in the meta-analysis and the circle the summary estimate. The light line is the confidence interval

Figure 3 shows the diagnostic accuracy of the studies distributed in three groups, PCR, LCR and Xpert MTB/RIF with a calculated confidence interval (CI) of 95% and a statistic continuity correction of 5%.

Within the studies that used PCR there was a wide heterogeneity by assessing the forest plot (Fig. 3). Because the low study quality of these studies, the different protocols and variants of PCR used, and the visual heterogeneity assessed in the forest plots, we decided not to proceed with a summary measure. Regarding LCR, only one study addressed this test.

**Fig. 4:**
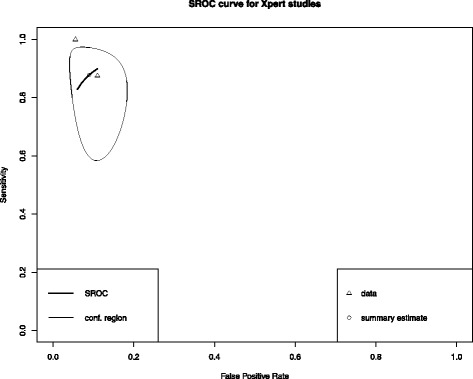
Summary Receiver-Operating Characteristic (SROC) curves for Xpert MTB/RIF assays

The two studies that used Xpert MTB/RIF showed good quality, and heterogeneity was further assessed by χ^2^ test. Table 2 shows the summary measures of these studies. We also plotted a SROC summary curve (Fig. 4). The summary sensitivity and specificity was 0·87 (0·66–0·96) and 0·91 (0·84–0·95) respectively.

**Table 2:** Summary measures of test accuracy of Xpert MTB/RIF studies

Test property	Summary measure of test accuracy ^a^ (95% CI)	Test for heterogeneity ^b^ *p*-value
Sensitivity	0·87 (0·66–0·96)	1
Specificity	0·91 (0·84–0·95)	0·27
Diagnostic odds ratio	58·16 (15·75–214·92)	0·43
LR+	9·66(4·12–19·2)	0·31
LR-	0·15(0·04–0·40)	0·36

^a^Random effects model ^b^ χ^2^ test for heterogeneity

## Discussion

Our systematic review indicates that Xpert MTB/RIF performance in urine samples for GUTB diagnosis showed sensitivity and specificity rates ranging from 0·83 to 0·95 and 0·79 to 0·99, respectively. While these results show acceptable diagnostic accuracy, a proper interpretation of results should take into account the wide confidence intervals of the outcome variables, several sources of heterogeneity, such as quality issues, different target genes, different protocols, and different number of specimens analyzed. It should be noted that Xpert MTB/RIF is a WHO recommended initial diagnostic test for all patients with sign and symptoms of TB [10].

Within the diverse protocols followed for PCR tests, possible sources of false positive and negative results are contamination and inhibition respectively. Contamination is usually caused by amplification products that get aerosolized and interfere with subsequent assays, this is especially true for nested PCR methods, in which repeated amplifications are done [27]. If not controlled, in a relatively short time, laboratory reagents, equipment, and even ventilation systems get contaminated with these products [27]. Metabolites, drugs, and other body fluid substances may produce inhibition of PCR; in urine, the most critical inhibitor is urea, which can lead to polymerase degradation in a concentration-dependent manner [28]. While doing PCR demands a controlled protocol to be followed carefully to avoid both inhibitors and contaminants, Xpert MTB/RIF does not face the same problem [29].

The pooled estimates of Xpert MTB/RIF studies in our analysis, showed satisfactory sensitivity and specificity: 0·87 and 0·91 respectively. However, only specificity had a narrow CI. Clinically it can be translated into the test correctly reporting 91% of patients without the disease as true negative. Because this meta-analysis only comprised two studies, further research is required to upgrade and get more precise pooled estimates. Additionally, positive predictive values (PPV) and negative predictive values (NPV) which depend on the prevalence of the disease should be reported for different clinical settings as they have more practical utility.

Information of drug resistance among patients with GUTB is lacking [1, 2, 30, 31]. In this context, none of the Xpert MTB/RIF studies analyzed rifampicin resistance, possibly because they were done in high-income countries, where multi-drug resistant (MDR) tuberculosis is not usually a concern. One study in China used a different test, GenoType® MTBDRplus, which evaluates isoniazid and rifampicin resistance, in an outpatient setting [32]. They found that one-third of their patients had drug-resistant GUTB, and one-fourth had MDR-GUTB. Subsequent Xpert MTB/RIF or GenoType® MTBDRplus studies must help characterize drug resistance epidemiology while evaluating the clinical utility of these tests in different MDR prevalence settings.

We identified two main limitations on studies, the reference standard and the number of specimens analyzed were not the same for all, and they did not report if test accuracy changed in patients with comorbidities.

In the studies that used positive urine culture as reference standard, there is chance that true positives cases with only clinical response after treatment were not detected but tagged as false positives; rendering lower specificity. Additionally, some studies analyzed more than one specimen per patient. While there is a consensus based on an expert opinion for using three consecutive morning samples for culture or NAATs, there is no study that addresses this particular issue [33] Increasing the number of specimens can increase sensitivity at the cost of lowering specificity. In addition,it is still unknown if this strategy is cost-effective.

Comorbidities within the populations studied were not reported in any of the articles selected. We were especially concerned about HIV patients, as it has been shown that accuracy of diagnostic tests varies in this population. In HIV-infected patients, those presenting with pulmonary TB typically have a paucibacillary disease [34, 35]. However, in tuberculous meningitis, two studies reported higher Xpert MTB/RIF accuracy in those patients infected with HIV [36, 37]. We suggest that authors consider HIV-status in future research in GUTB patients.

### Strengths and weaknesses of the study

We identified some weaknesses in our review. We could not assess male and female genital tuberculosis through this review because they lack a clear reference standard and thus, there would be a high concern of bias in the analysis [20]. We did not evaluate the effect of adding NAATs to other tests, and the net effect of NAATs on positive predictive values or negative predictive values since these depend on the prevalence of the disease. Furthermore, we could not explore the effect of issues such as expertise with NAATs equipment, and laboratory infrastructure because of poor reporting.

Our study has several strengths: an exhaustive search strategy (including conference abstracts, and no language restriction), with more than 2000 patients evaluated and a deep critical quality appraisal, exploring heterogeneity in accordance with published guidelines, and following PRISMA recommendations for systematic reviews [22].

### Limitations

The lack of quality reporting in diagnostic interferes with critical appraisal and replication of studies [38]. We encourage authors to follow the Standards for Reporting Diagnostic Accuracy (STARD) in order to achieve transparency, completeness and excellence of reporting [39]. The STARD was updated in 2015 [40], and comprises a list of 30 essential items for high-quality diagnostic accuracy studies; it has also incorporated evidence about sources of bias and variability in diagnostic accuracy.

## Conclusion

GUTB is an overlooked disease, an unspecific clinical picture and limited conventional tests account for this problem. Diagnosis is difficult and frequently delayed, leading to major impact on the urinary tract system.

All PCR studies were highly heterogeneous as they involved different gene targets, different protocols and had several quality concern issues. Consequently, no meta-analysis could be done. Xpert MTB/RIF studies, in contrast, were high quality reported and were not heterogeneous. Our analysis showed a favorable specificity. Studies with high-quality reporting are urgently needed to improve diagnosis of GUTB.

## Additional file

Additional file 1: Complete search strategy and details on quality evaluation. (DOCX 18 kb)

## Abbreviations

EPTB: Extrapulmonary tuberculosis
GUTB: Genitourinary tuberculosis
LCR: Ligase chain reaction
MDR: Multi-drug resistant
NAAT: Nucleic acid amplification test
NPV: Negative predictive values
PCR: Polymerase chain reaction
PPV: Positive predictive values
QUADAS-2: Quality Assessment Tool for Diagnostic Accuracy Studies
SROC: Summary receiver-operating curve
STARD: Standards for Reporting Diagnostic Accuracy
TB: Tuberculosis
UGTB: Urogenital tuberculosis
WHO: World Health Organization
