# Supplementary material for: Diagnostic accuracy of nucleic acid amplification tests (NAATs) in urine for genitourinary tuberculosis: a systematic review and meta-analysis
Source: BMC Infect Dis. 2017 Jun 5;17:390. doi: 10.1186/s12879-017-2476-8 (PMC5460328; doi:10.1186/s12879-017-2476-8)
Supplement: Additional file 1: — Complete search strategy and details on quality evaluation. (DOCX 18 kb) [file 12879_2017_2476_MOESM1_ESM.docx]

**Additional file 1**

**APPENDIX 1**

**Complete Search Strategy**

**PubMed:**

("Nucleic Acid Amplification Techniques"[Mesh] OR "Polymerase Chain Reaction"[Mesh] OR "Real-Time Polymerase Chain Reaction"[Mesh] OR "Reverse Transcriptase Polymerase Chain Reaction"[Mesh] OR "Multiplex Polymerase Chain Reaction"[Mesh] OR "genexpert”[tw] OR "genotype”[tw])

AND

(("Tuberculosis, Urogenital"[Mesh] OR "Tuberculosis, Renal"[Mesh]) OR ("Urogenital System"[Mesh] AND "Tuberculosis"[Mesh]) OR “Genitourinary tract tuberculosis”[tw] OR (“Extrapulmonary tuberculosis”[tw] AND “Urine”[tw]))

**Embase (a través de los comandos de Ovid):**

1. exp *urogenital tuberculosis/

2. polymerase chain reaction/ or exp *gene amplification/ or exp *nucleic acid analysis/

3. exp kidney tuberculosis/

4. Urogenital system.mp.

5. exp extrapulmonary tuberculosis/

6. exp urine/

7. 5 and 6

8. exp *female genital system/

9. Genexpert.mp.

10. (MTBDR* or "Genotype MTBDR*").mp.

11. tuberculosis.mp. or Mycobacterium tuberculosis/

12. 4 and 11

13. 8 and 11

14. 2 or 9 or 10

15. 1 or 3 or 7 or 12 or 13

16. 14 and 15

**Lilacs:**

(tw:(tuberculosis urogenital)) OR (tw:(tuberculosis genitourinaria)) AND (tw:(pcr)) OR (tw:(pruebas amplificación nucleica)) OR (tw:(genexpert)) AND (instance:"regional") AND ( db:("LILACS"))

**Scopus:**

TITLE-ABS-KEY (genitourinary tuberculosis ) AND TITLE-ABS-KEY ( Nucleic acid amplification test)

**Web of Science:**

Tema: (Nucleic acid amplification test) AND Tema: (Genitourinary Tuberculosis)

**Cochrane Library:**

Genitourinary Tuberculosis

**APPENDIX 2**

**QUADAS-2 rules and interpretation**

We use "patients" below with the understanding that studies in this review may be evaluating patient specimens.

**Domain 1: Patient selection**

**Risk of bias: Could the selection of patients have introduced bias?**

**Signaling question 1: Was a consecutive or random sample of patients enrolled?**

We will score 'yes' if the study enrolled a consecutive or random sample of eligible patients; 'no' if the study selected patients by convenience, and 'unclear' if the study did not report the manner of patient selection or was not clearly reported.

**Signaling question 2: Was a case-control design avoided?**

We will score 'yes' if the study enrolled only GUTB suspected patients; 'no' if the study enrolled GUTB confirmed patients; and 'unclear' for all other scenarios or if it was not clearly reported.

**Signaling question 3: Did the study avoid inappropriate exclusions?**

An inappropriate exclusion might occur if, after the laboratory technician runs the index and reference tests, he or she does not record the test results in the study. This might occur if there were resource constraints as one might find in practice, but we do not expect this to occur in the research studies included in this review. We will score 'yes' for all studies, as we do not anticipate inappropriate exclusions.

**Applicability: Are there concerns that the included patients and setting do not match the review question?**

We will judge 'low' concern if the selected specimens match the review question, which reflects the way the test will be used in practice. We will judge 'high' concern if the selected specimens or isolates do not represent those for which the test will be used in practice, such as in individuals who are not suspected of having GUTB. We will judge 'unclear' concern if we cannot tell.

**Domain 2: Index test**

**Risk of bias: Could the conduct or interpretation of the index test have introduced bias?**

**Signaling question 1: Were the index test results interpreted without knowledge of the results of the reference standard?**

We will score this question 'yes' if the reader of the assay was blinded to results of reference tests. We will score 'no' if the reader of the assay was not blinded to the results of reference tests. If the specimens were from a biobank comprised of specimens with known GUTB and the identity of these specimens was known to the assay reader, we will also answer 'no'. We will score 'unclear' if it was not stated in the paper or if the authors failed to answer this question.

**Applicability: Are there concerns that the index test, its conduct, or its interpretation differ from the review question?**

As our review question is broad, variations in test technology, execution or interpretation may affect estimates of the diagnostic accuracy of a test. We will first cluster similar type of tests together and then analyze the variation between them. We will judge these issues to be of 'low' concern if the specifications and gene target are the same. If not, we will judge these issues to be ‘high’ concern.

**Domain 3: Reference standard**

**Risk of bias: Could the reference standard, its conduct or its interpretation have introduced bias?**

**Signaling question 1: Is the reference standard likely to correctly classify the target condition?**

Culture of AFB is currently considered the gold standard for diagnosing GUTB, however, it is sensitivity is not high in urine specimens, tissue specimens, and semen, therefore many studies use a combination of other findings as the reference standard: either a positive culture or a positive microscopy; or a combination of clinic characteristics and resolution of signs or symptoms after appropriate treatment. Whenever the reference standard of the study appraised is defined as any of above, we will answer ‘yes’ ; when the reference standard is different, we will answer ‘no’.

**Signaling question 2: Were the reference standard results interpreted without knowledge of the results of the index test?**

We will score 'yes' if the blinding was explicitly stated, or it was clear that the reference test was performed at a separate laboratory, or performed by different people, or both. We will score 'no' if the study stated that the reference standard result was interpreted with knowledge of the index test result. We will score 'unclear' if it was not stated in the paper or if the authors failed to answer this question.

**Applicability: Are there concerns that the target condition as defined by the reference standard does not match the question?**

We judge applicability to be of 'low concern' for all studies that use the above-stated reference standard, but to be of ‘high concern’ by those studies that have defined their own reference standards.

**Domain 4: Flow and timing**

**Risk of bias: Could the patient flow have introduced bias?**

**Signaling question 1: Was there an appropriate interval between the index test and reference standard?**

We expect the reference standard test to be undertaken at the same time as the index test (ie each performed on a paired sample for the majority of studies). However, we expect some studies to include specimens from patients who have received a reference test on an earlier sample. We will answer 'yes' if the tests were paired or were separated by a few days. We will answer 'no' if reference and index tests were not done on paired samples and were separated by several months. We will score 'unclear’ if it was not stated in the paper or if the authors failed to answer this question.

**Signaling question 2: Did all patients receive the same reference standard?**

We will answer 'yes' if the same reference standard was applied to all patients or a random sample of patients, 'no' if the reference standard was only applied to a selective group of patients and 'unclear' if it was not stated in the paper or if the authors failed to answer this question.

**Signaling question 3: Were all patients included in the analysis?**

We will determine the answer to this question by comparing the number of participants enrolled with the number of patients included in the two-by-two tables. We will note if the authors report the number of indeterminate assay results.

We will score 'yes' if the number of participants enrolled was clearly stated and corresponded to the number presented in the analysis or if exclusions were adequately described. We will score 'no' if there were participants missing or excluded from the analysis and there was no explanation given. We will score 'unclear' if not enough information was given to assess whether participants were excluded from the analysis.

**Table S1: Detailed results of QUADAS-2 evaluation**

| **Study** | **RISK OF BIAS** | | | | **APPLICABILITY CONCERNS** | | |
| --- | --- | --- | --- | --- | --- | --- | --- |
|  | **PATIENT SELECTION** | **INDEX TEST** | **REFERENCE STANDARD** | **FLOW AND TIMING** | **PATIENT SELECTION** | **INDEX TEST** | **REFERENCE STANDARD** |
| Khan et al | ☺ | ? | ? | ☺ | ☺ | ? | ☺ |
| Garcia-Elorriaga et al | ☺ | ☹ | ☹ | ☹ | ☺ | ☹ | ☹ |
| Khosravi et al | ☺ | ☹ | ☹ | ☺ | ☺ | ☹ | ☺ |
| Raghavendran et al | ☺ | ☹ | ☹ | ☺ | ☺ | ☹ | ☹ |
| Hemal et al | ☺ | ☹ | ☹ | ☹ | ☺ | ☹ | ☹ |
| van Vollenhoven et al | ☺ | ☹ | ☹ | ☺ | ☺ | ☹ | ☺ |
| Moussa et al | ☺ | ☹ | ☹ | ☺ | ☺ | ☹ | ☺ |
| Moussa et al | ☺ | ☹ | ☹ | ☺ | ☺ | ☹ | ☺ |
| Gamboa et al | ☺ | ☹ | ☹ | ☺ | ☺ | ☹ | ☺ |
| Hillerman et al | ☺ | ☺ | ☺ | ☺ | ☺ | ☺ | ☺ |
| Tortoli et al | ☺ | ☺ | ☺ | ☺ | ☺ | ☺ | ☺ |

☺Low Risk ☹High Risk ? Unclear Risk
